# Differential Toxicity of Ionic Silver and Silver Nanoparticles: A Meta-Analysis of Ecotoxicological Studies

**DOI:** 10.3390/toxics14010028

**Published:** 2025-12-26

**Authors:** Esra Karaman, Deniz Boz Eravci, Selim Latif Sanin, Vugar Ali Turksoy

**Affiliations:** 1 Department of Environmental Engineering, Institute of Science, Hacettepe University, 06800 Ankara, Türkiye; esrakaraman@hacettepe.edu.tr (E.K.); sanin@hacettepe.edu.tr (S.L.S.); 2 Department of Occupational Health and Safety, Institute of Science, Ankara Yıldırım Beyazıt University, 06010 Ankara, Türkiye; denizbozdb@gmail.com; 3 Department of Public Health, Faculty of Medicine, Yozgat Bozok University, 66100 Yozgat, Türkiye

**Keywords:** silver, toxicity, meta-analysis, ionic silver, nanoparticulate silver, environmental toxicology

## Abstract

The literature on the toxicity of silver metal has increased in recent years. However, these studies differ in terms of silver forms, test organisms and exposure times. This makes it difficult to compare results and hinders the development of reliable guidelines on silver toxicity. This study presents a systematic meta-analysis to clarify the comparative toxicity of AgNO_3_ and AgNPs on a wide range of biodiversity species, including prokaryotes, unicellular eukaryotes, invertebrates, fish, and terrestrial organisms. We screened 1117 studies published between 1945 and 2024, systematically applied the screening criteria and analyzed 28 data sets from 11 studies that met the eligibility and data quality criteria. The findings demonstrate that AgNO_3_ exhibits higher toxicity than AgNPs in most cases, and this effect is particularly pronounced in various organisms. Furthermore, exposure duration is found to be a critical determinant, creating significant differences in both short-term (from 3 h) and long-term (96 h and above) exposures. This study demonstrates that silver toxicity is dependent on forms of silver, and shaped by exposure dose, time-dependent and organism types. A key point in this study is that the evidence base covers the years representing the broadest temporal scope among comparable studies. The results provide a quantitative synthesis of the existing literature, allowing for the identification of generalizable trends regarding the ecotoxicological effects of silver and shed light on the environmental risk assessment processes of silver forms.

## 1. Introduction

Silver metal has been widely used in various sectors and applications such as biomedical, air and water purification, cosmetics and textiles, and automotive throughout human history due to its broad-spectrum antimicrobial properties [1,2,3,4]. Silver metal is toxic to both the human body and living organisms in water and soil [5,6,7,8,9]. Even at low concentrations, silver is known to inhibit the growth, reproduction and survival of sensitive aquatic species such as algae, daphnia and fish [6,10,11,12]. After being taken into the cell, silver binds to enzymes and protein structures and disrupts cell functions [7,13,14].

Silver metal is used in various forms such as metallic silver nanoparticles, silver chloride particles, silver impregnated zeolite powder and activated carbon materials, polymer silver nanoparticle composites, silver titanium dioxide composite nanoparticles, and silver nanoparticle-coated polymers [1,15]. All of these forms have different levels of antimicrobial activity depending on the release of silver ions. However, it is known that silver in nanoparticle form may exhibit additional antimicrobial properties compared to solution or ionic silver [3,16]. Among these forms, the toxicological effects of AgNO_3_ and AgNPs in particular have been the subject of intensive research [17,18]. AgNPs are subject to processes such as sedimentation, agglomeration, dissolution and surface transformations in the environment, and these processes make it difficult to determine the actual source of toxicity [19,20,21].

The toxic effects of AgNPs are thought to be largely due to the Ag^+^ ions they release by dissolving [22,23]. However, some studies suggest that the nanoparticle itself can show independent toxic effects by physically disrupting the cell membrane or by generating reactive oxygen species (ROS) [24,25]. When silver ions are taken into the cell, they interact with enzymes and protein structures, causing disruption of cellular functions [24,26].

Studies on different organisms and test systems have revealed contradictory findings. For example, while studies on *Daphnia magna* have shown that AgNO_3_ is more toxic [27], AgNPs have been reported to have a more dominant effect on some algae species [28,29]. The reasons for these contradictory findings may include factors such as differences in experimental conditions, types of organisms used, nanoparticle size, coating type, test duration, and environmental chemistry [30,31].

Studies on toxicology usually assess lethal concentration, effects on development or reproduction [32,33,34]. Evaluating the toxicology of rapidly expanding products such as nanomaterials on different species and under different conditions is time and cost-consuming. On the other hand, since nanoparticles constitute a large and uncertain environmental problem area, the need for rapid and sensitive monitoring tools is increasing. In recent years, the use of meta-analyses has increased to provide quantitative synthesis of ecological and toxicological data [35,36,37].

At this point, the meta-analysis method is an effective tool for bringing together quantitative data obtained from different studies and revealing general trends [38]. The increasing data accumulation in the nanotoxicology literature in recent years makes the applicability of this method possible [39]. In addition, there is still limited data in terms of ecosystem representation in the field of nano-ecotoxicology, and meta-analysis allows for evaluating this gap in a structured way [40].

The aim of this study is to comparatively analyze the toxic effects of ionic silver (AgNO_3_) and silver nanoparticles (AgNPs) on various organisms. In this study, the comparative toxic effects of ionic and nanoparticle silver were evaluated using the meta-analysis method based on toxicity data obtained from various organisms. Although the species included in the study have different physiological and trophic characteristics, they do not represent the entire ecosystem. Therefore, the generalization of the results should be interpreted only in the context of the ecotoxicological responses of the analyzed organisms.

To this purpose, we conducted a meta-analysis, including a systematic review of the literature, to compare the toxicity of the most commonly used ionic and nanoparticulate forms of silver and to assess the impact of this toxicity. The study quantitatively evaluates the complex and sometimes contradictory literature on silver toxicity and demonstrates that the meta-analysis method is an applicable tool for use in environmental research. This study aims to provide a systematic and statistical overview of the contradictory results in the field by comparatively evaluating the toxicity findings related to ionic and nanoparticle silver in the literature with the meta-analysis method.

In conclusion, this study systematically synthesizes existing data to support risk assessment and policy-making efforts on silver toxicity, providing an inclusive method for existing contradictory results.

## 2. Materials and Methods

In this study, studies comparing the toxicity of two different forms of silver on various organisms were quantitatively evaluated by meta-analysis. The set of techniques used to combine the results of different studies to create a single and more precise effect in a study is called meta-analysis [41,42]. Meta-analysis is a method that aims to make quantitative findings from independent studies comparable by converting them to a common unit of measurement. Effect size, used in this process, is a standardized measure that indicates the degree to which a variable creates a significant difference under experimental conditions. To calculate effect sizes reliably, studies must adequately report basic statistics such as sample size, central tendency, and variance. The data obtained in this study were combined within the framework of a random-effects model, and the reliability of the results was assessed using heterogeneity analyses. (Cochran’s Q, I^2^) [41,43]. Furthermore, variables known in the literature that contribute to the heterogeneity of toxicity results, such as exposure duration and ecosystem type (aquatic, terrestrial), were considered in the subgroup analyses. The package program Comprehensive Meta-Analysis (CMA) (https://www.meta-analysis.com/ (accessed on 15 May 2025) (USA)) V3.7 was used for meta-analysis in the study. The systematic review and meta-analysis process was conducted in accordance with the Preferred Reporting Items for Systematic Reviews and Meta-Analyses (PRISMA) 2020 guidelines [44,45], and the study was registered in the International Prospective Register of Systematic Reviews (PROSPERO) database (ID: 1054634) [46]. This study is a combination of the following steps:

### 2.1. Search Strategy and Inclusion/Exclusion Criteria for Meta-Analysis

A systematic search was conducted using the “Web of Science [v.5.35]” search engine and ‘Web of Science Core Collection’ database. This database was chosen because of its broad coverage of toxicology, environmental science and nanomaterial-related studies. Advanced Search” search option and ‘TS’ field tag was used. The keywords used were selected to include the most comprehensive results relevant to the research problem and to represent commonly used concepts. All studies in which ion and nanostructured silver metal were included in the same study represent the population of the study. The search set created is as follows: “TS = ((nanoparticle silver OR nano particle silver) AND ionic silver)”. The search set was created with the options “All years”, “All languages”, “All document types” as year, language and document type, respectively. Given that toxicity trends in studies may change over time and because the goal was to provide comprehensive results, no year limitation was applied. The literature review was conducted from a database of scientifically accepted studies. The screening and selection process was conducted in accordance with the internationally accepted PRISMA guidelines for systematic reviews and meta-analyses. In order to increase the transparency and methodological accuracy of our meta-analysis, the protocol was registered before the study and published in the PROSPERO database (ID Number:1054634).

Toxicity databases (e.g., ECOTOX, ECHA) were excluded because, in most cases, in addition to effect concentrations, statistical parameters required for meta-analysis (e.g., confidence interval, standard deviation, *p*-value) are missing, and experimental conditions are not given in sufficient detail. Furthermore, these sources lack adequate reporting of contextual factors such as organism type, test environment, and exposure time, limiting the comparability of data obtained from studies. In addition, quasi-experimental studies, randomized controlled trials, and case-control studies were not included, so no additional evaluation was made for the publication quality of the studies. Studies from databases searched by keywords were reduced according to inclusion and exclusion criteria. The reasons for inclusion and exclusion criteria are listed as follows:

- The primary screening criterion was based on study type and language. Studies not written in English were excluded, as were encyclopedias, reviews, conference abstracts, book chapters, and abstracts of articles that were not available in full. All years for which the database was searchable (1945–2024) were included. So, this criterion ensured the inclusion of studies that were methodologically reliable and accessible for full-text evaluation, improving the transparency and reproducibility of the screening process.
- The secondary exclusion criterion is based on the methodology of research studies. Studies showing the toxicity of nano and ionic silver on the same species in the same study were included. This allowed for a comparative assessment of toxicity differences within the same biological context. Including studies that assessed both forms within the same biological context allowed for a more controlled and meaningful comparison of ionic versus nanoparticulate silver toxicity, addressing potential confounding factors.
- The third criterion of exclusion is related to the units of the results. The study included toxicity value and effective concentration dose–response curves. Additionally, studies lacking standard deviation values in toxicity results were excluded. This criterion is essential for the reliability of effect size calculations to be used in statistical analysis. Requiring compatible units on data and the presence of standard deviation ensured that effect sizes could be calculated accurately, which is essential for maintaining the statistical validity of the meta-analysis.

### 2.2. Data Extraction and Coding

After applying the primary screening criteria to the literature review, all the included studies were transferred to a Microsoft Excel spreadsheet in the form of a list. Columns containing descriptive characteristics of the studies (DOI number, date, authors, type of study) and secondary screening criteria were created. At this stage, only clearly reported toxicity measures suitable for direct comparison were considered to ensure the scientific validity of the data to be included in the meta-analysis. According to these criteria, the review was conducted by 3 researchers on the full text of the studies. The evaluation process between the 3 researchers was conducted independently to increase methodological consistency, and coding differences were resolved by mutual agreement. Studies that met the tertiary screening criteria were saved together with full text and supplemental data files using the Mendeley Cite plugin, and traceability was ensured. Finally, all exclusion criteria were evaluated, and the studies to be included in the meta-analysis were determined. The entire selection process was documented in accordance with the PRISMA workflow and regularly reviewed to ensure the reproducibility of the registered dataset. The process of exclusion and inclusion continued until the researchers reached a consensus, and was monitored by an independent reviewer. This aim is to minimize inter-coder bias and maintain the objectivity of selection decisions. In the final stage, a data coding system was used to use the results obtained from the included studies in the meta-analysis program. Authors, year, initial concentration, particle size, toxicity data, test results such as temperature, pH, t and *p* were recorded. These variables were obtained from studies that were meaningful and adequately reported for meta-analytic subgroup analyses and heterogeneity assessment. Relevant data from each study were recorded, including author and publication year. During the coding process, all data were double-checked by a secondary reviewer for verification.

### 2.3. Meta-Analysis

The standardized mean difference was calculated to compare the toxic effect of the nanoparticle form of silver compared to the ionic form. The standardized mean difference is a statistical measure of effect size that expresses the mean difference between two groups on a standardized scale, considering the standard deviations of the groups. Comparison of two groups, time-points or exposures (includes correlations) was selected to calculate the effect size in the CMA program. First group data refers to AgNPs toxicity values, and second group data refers to AgNO_3_ toxicity values. In this study, toxicity values (EC50, LC50 and LD50) reported across the included publications were pooled under a unified metric referred to as the toxicity value (TV); all publications included in this study reported only EC50 values. Because all endpoints were converted into standardized effect sizes (Hedges g), pooling these measures did not influence the proportional structure or the overall direction of the analysis. Effect size magnitudes were interpreted as small effect if 0.2 ≤ standardized mean difference < 0.5, medium effect if 0.5 ≤ standardized mean difference < 0.8 and large effect if 0.8 ≤ standardized mean difference [41,47]. The direction of effect was chosen as “Automatic”. + results mean that the effect of the first group is larger; − results mean that the effect of the second group is larger. When comparing two groups, the program takes into account the mean, standard deviation and sample size in each group [46,47].

#### 2.3.1. Heterogeneity Testing and Model Selection

The heterogeneity test of the study was evaluated based on Q-value, df (Q), *p*-value and I^2^ values. Heterogeneity is explained by a test of the null hypothesis that the true effect size is the same in all studies and that 100% of the variation in observed effects is due to sampling error. Here, if *p* < 0.05, the null hypothesis is rejected, meaning that effect sizes are heterogeneous. In addition, an I^2^ value higher than 75% indicates high heterogeneity between studies. In case of heterogeneous effect values, the random effect model is used [48,49]. This approach allows for more generalizable results to be obtained by considering the diversity in toxicity responses of different experimental environments and organisms. Standardized mean differences were calculated using Hedges’ g with small-sample bias correction, as implemented in the Comprehensive Meta-Analysis software (V3.7 version, USA). Between-study heterogeneity (τ^2^) in the random-effects model was estimated using the DerSimonian–Laird (DL) method, which is the default setting in CMA.

#### 2.3.2. Subgroup Analysis

Subgroup analyses were conducted to identify potential sources of heterogeneity, with a particular focus on exposure time and ecosystem type (aquatic and terrestrial). Both variables were selected for subgroup analysis because they were consistently reported across a substantial number of primary studies. By stratifying the dataset according to exposure periods and ecosystem categories, the analysis aimed to clarify how temporal factors and environmental conditions influence the toxicity of silver compounds. This approach allows for testing whether effect sizes differ across exposure durations and ecosystem types, thereby providing a better understanding of the sources of heterogeneity.

#### 2.3.3. Publication Bias

In the study, Begg and Mazumdar used rank correlation statistics to determine whether there was any publication bias. Accordingly, Kendall’s Tau coefficient is calculated, and if this coefficient is close to 1 and the two-tailed *p*-value does not create a significant difference, that is, if the *p*-value is greater than 0.05, it is interpreted as the absence of publication bias [50]. This test is widely used to assess the presence of systematic bias in small-scale studies. To evaluate the significance of publication bias, funnel plots were applied to make rough qualitative observations by visual inspection of their symmetry [51]. The symmetrical funnel plot indicates that small-scale studies with both positive and negative results were represented evenly, and therefore, unpublished or statistically insignificant studies were not systematically excluded.

## 3. Results

### 3.1. Literature Search and Inclusion Criteria Results

As a result of the literature search, a total of 1117 studies were found. After applying the primary screening criteria (study type and language), publications other than English, reviews, encyclopedias, conference abstracts, book chapters, and articles available only as abstracts were excluded, resulting in the elimination of 103 studies. Following this step, 1014 studies were evaluated. Secondary screening criteria based on research methodology excluded studies that did not provide comparative toxicity data on both AgNO_3_ and AgNPs in the same species. This elimination resulted in the exclusion of 639 studies, and 375 were eligible for full-text review. The last criterion, based on outcome measures and statistical adequacy, excluded studies that did not report toxicity values based on dose–response curves or did not provide statistical parameters such as standard deviation/variance. 364 studies were eliminated at this stage, leaving a total of 11 studies that met the inclusion criteria for the meta-analysis. The included studies encompassed diverse organismal groups, including microorganisms, algae, crustaceans, and fish. Furthermore, data from both aquatic and terrestrial ecosystems were included in the meta-analysis, reflecting a diversity of exposure times and experimental conditions. This allows for comparisons of the results across organisms and ecosystems. This number was determined based on the limited number of experimental studies comparing both ionic and nanoparticulate silver forms on the same organism and the requirement to meet the statistical parameters required for meta-analysis (sample size, mean, standard deviation).

**Figure 1:**
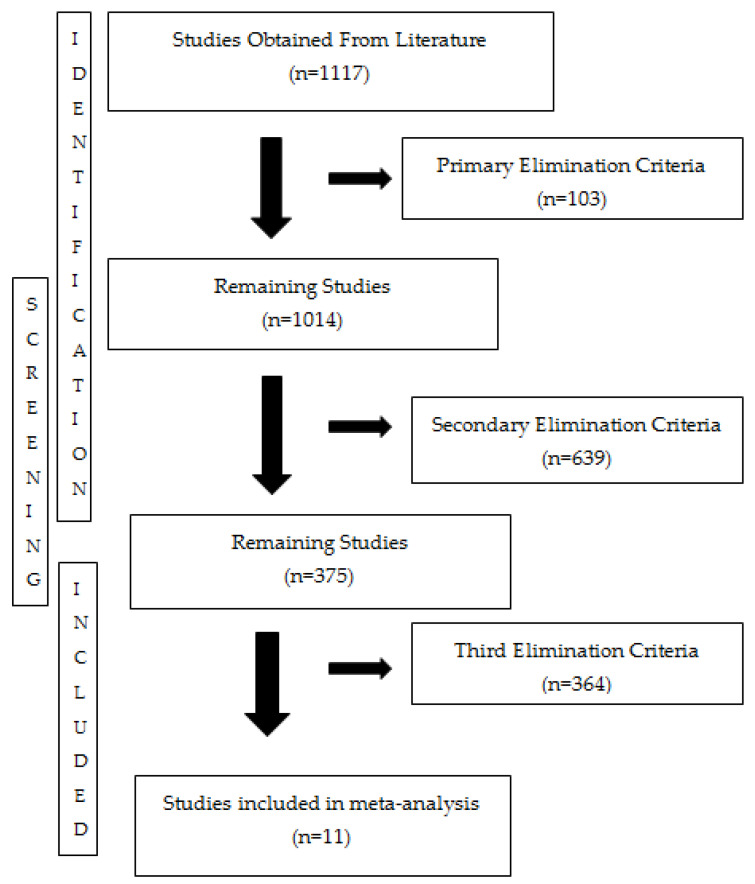
PRISMA (Preferred Reporting Items for Systematic Reviews and Meta-Analysis) flowchart of meta-analysis. It outlines the steps followed during the systematic literature search and shares detailed information about the results obtained, helping to ensure the process is transparent and can be reliably reproduced by other researchers.

In this meta-analysis study, since the physicochemical properties of the silver form (size, shape, coating, oxidation state, etc.) were not reported in sufficient detail in each publication, these variables could not be included separately in the analytical model. This was considered a limitation of the scope of the analysis. This precluded subgroup analyses based on these variables; however, the analysis provided sufficient methodological diversity to enable meaningful comparisons based on form type (ionic vs. nanoparticulate) and duration of exposure. Despite this limitation, all studies included in the meta-analysis produced meaningful and generalizable findings for assessing toxicity differences at the toxicity values, as they included comparative experimental data and provided key toxicological parameters. The PRISMA Flow Chart showing the study phases is presented in Figure 1. This flowchart reflects the preferred reporting standards for systematic reviews and meta-analyses [44].

PRISMA flow chart shows the stages of identifying, screening, and including the literature in the study. Additionally, this three-stage evaluation process aimed to ensure that the data used in the study had a high degree of integrity in terms of both content and statistical relevance. Reasons for exclusion and the justification of the criteria are presented in detail in the Section 2.

### 3.2. Data Extraction Results and Coding

The total sample size from the 11 studies included in the meta-analysis was 28, corresponding to 28 effect sizes reported across different organisms, endpoints, and exposure conditions. “Sample size” here refers to the number of independent toxicity data included in the meta-analysis for each study in this context. If different organisms, exposure times, or experimental conditions were tested in the same study, each was considered a separate data point. The studies covered different organisms, such as microorganisms, algae, crustaceans, and fish, and included data from both aquatic and terrestrial ecosystems.

Exposure times varied across the included studies, and experimental conditions (e.g., temperature, pH, and medium composition) varied from study to study. The basic characteristics of the included studies are systematically summarized by study name (author and year), sample size, organism tested, exposure time, ecosystem type, and experimental conditions, and are presented in Table 1. The data in Table 1 reveals the diversity of organisms included in the meta-analysis, the diversity of experimental conditions, and particularly the wide distribution of exposure durations. This is one of the key parameters that justify the subgroup analyses employed in the study. Although the number of studies included is limited, the representation of different organism types and the diversity of observed toxic effects increase both the comparability of the data and their generalizability to different environmental contexts.

Table 1 provides a general summary of the studies included in this meta-analysis. The studies are quite diverse in terms of the target organisms used in toxicity tests. The organisms examined include bacteria (*Escherichia coli*), single-celled algae (*Chlorella autotrophica*, *Dunaliella salina*, *Pseudokirchneriella subcapitata*), invertebrates (*Ceriodaphnia dubia*, *Daphnia magna*, *Physa acuta*, *Caenorhabditis elegans*), and vertebrates (*Danio rerio*, *Cyprinus carpio*, *Oryzias latipes*). This diversity allows for the assessment of differences in toxic responses among organisms at different trophic levels, thus enhancing the generalizability of analyses across environmental contexts. Exposure durations vary between studies, covering short- and medium-term toxic exposures ranging from 3 h to 96 h. In studies, toxicological effect measures (e.g., EC50) for differently sized AgNPs and AgNO_3_ were reported separately. In these cases, each size was evaluated in its own experimental condition and included in the analysis as an independent effect size. Each toxicity value was evaluated as an independent effect size, considering the corresponding experimental conditions and organism type. Temperature values typically range from 18 °C to 35 °C, while pH values are maintained within a narrow range of 6.8 to 8.3. However, pH and t values are not provided simultaneously in every study. This supports the analytical comparability of TV data across different studies, as it demonstrates that they are obtained within a certain range of environmental consistency. Overall, this table provides a foundation for comparative analyses in terms of organism types and exposure durations.

### 3.3. Meta-Analysis

#### 3.3.1. Heterogeneity Testing and Model Selection

The heterogeneity test was performed by calculating the statistical values of the weighted sum of squares Q (2013.040), df (Q) (27), *p* (0.000) and I^2^ (98.659). Since the *p*-value in the study was (0.000); *p* < 0.05, the effect sizes of the studies were heterogeneous. The I^2^ value in the study was 98.659, and this indicates high heterogeneity of the meta-analysis results. A significant heterogeneity value indicates that variables that may be specific to the study, such as exposure time, organism type, test conditions (pH, temperature) and particle properties, differ significantly. In this study, a random effects model was adopted to incorporate heterogeneity between studies. The random effects model operates on the assumption that each study may have a different true effect size and provides more reliable and generalizable results, especially in cases of high heterogeneity. A random effects model was applied for a total of 28 effect sizes included in the meta-analysis, and the overall effect size was calculated as 3.268 (95% CI = 1.805–4.731; *p* = 0.000). These results suggest that the variability among studies should be taken into consideration and necessitate the application of subgroup analyses to identify potential sources of heterogeneity.

**Figure 2:**
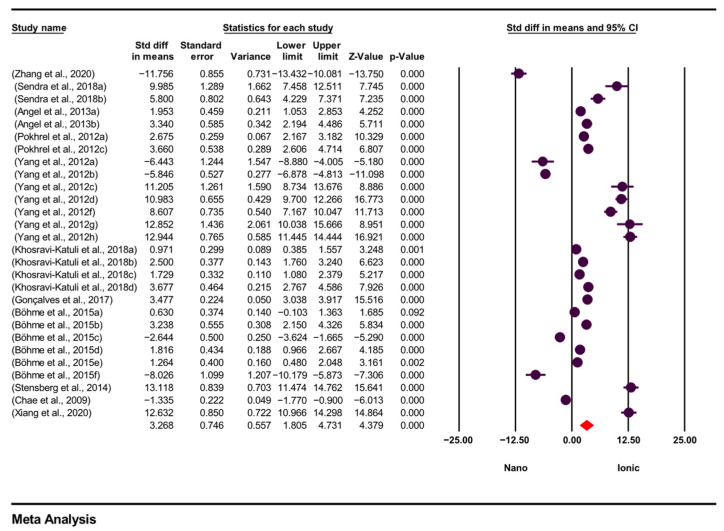
Effect size (SMD), confidence interval and significance values of studies and forest plots of studies (n = 28) included in meta-analysis. “SMD” (Standardized Mean Difference) expresses the difference in toxic effects between the experimental and control groups in standard deviation units. The “Standard Error” and “Variance” columns show the statistical reliability of this value. “Lower Limit” and “Upper Limit” represent the 95% confidence interval, and the true effect size is likely to lie within this interval. “Z-Value” tests whether the effect size is different from zero, while “*p*-Value” indicates the statistical significance of this difference. The combined effect size values obtained using the fixed and random effect models are presented in the “Model Type” rows. Forest plot showing effect sizes (std diff in means; SMD) and 95% confidence intervals (CI) from 28 studies comparing toxicity levels Nano = AgNPs TV and Ionic = AgNO_3_ TV. Effect sizes were estimated with a random-effects model that takes into account between-study variation. Positive values indicate higher TV for nanoparticulate silver, thus indicating lower toxicity compared to ionic silver. Statistical significance was set at *p* < 0.05. CI = confidence interval; SMD = standardized mean difference; TV = toxicity value.

When interpreting the results of the meta-analysis, the results calculated in the random effects model were used to express the effect size of the study. This meta-analysis found a significant difference in toxicity levels between ionic and nanoparticle forms of silver. The standardized effect size for the random model obtained is positive, indicating that the TV of AgNPs is higher than AgNO_3_, thus indicating that ionic silver appears to be more toxic overall, but the variation between studies is very high. The standardized effect size, related statistical error and confidence intervals and significance levels of each study included in the meta-analysis are presented in Figure 2.

This analysis allows for evaluating the contribution of each of the studies included in the meta-analysis to the total effect size separately. The results obtained show a generally significant effect size in terms of the toxic effects of ionic and nanoparticle silver. The standardized mean difference (SMD) makes it possible to compare the effect between two groups independently of the measurement units; in this respect, it allows the integration of TV from different studies in a common analysis. These findings provide an important scientific basis for the main purpose of the study, which is to compare the toxic effects of the two forms of silver. The visual representation of the findings is given in Figure 2, with a forest plot presenting the effect sizes and confidence intervals of individual studies together. A 95% prediction interval was also calculated to reflect the expected range of true effects in comparable future studies.

The total standardized difference in mean representing all studies is in the + direction. This means that the values for the first data set (AgNP TV) are greater than the values for the second data set (AgNO_3_ TV). The confidence interval for all studies coincides with the average effect size. Since the effect size according to the standardized mean difference in all studies (fixed: 1.944; random: 3.268) is >0.8, this effect is interpreted as large.

#### 3.3.2. Subgroup Analysis Results

Due to the high heterogeneity of the meta-analysis, we conducted a predefined subgroup analysis, focusing on two key factors: exposure time and the type of ecosystem.

**Figure 3:**
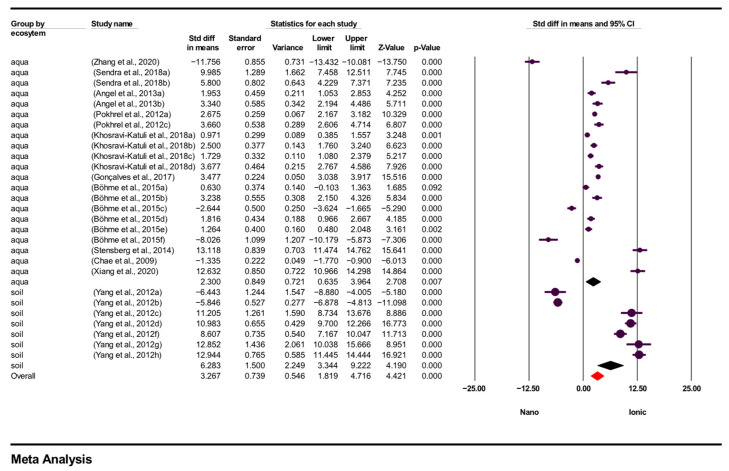
Subgroup analysis according to type of ecosystem and forest plots (n = 28). CI = Confidence Interval; *p* = *p* < 0.05; aqua = aquatic; soil = terrestrial; Overall = Total mean size between studies; Nano = AgNPs TV; Ionic = AgNO_3_ TV.

First, in the analysis by ecosystem type, studies were divided into aquatic and terrestrial groups. According to the results of the subgroup analysis based on these ecosystems overall effect size was 3.267 (95% CI = 1.819–4.716; *p* = 0.000), indicating that ecosystem type made a statistically significant difference in the results. In aquatic ecosystems, effect size was 2.300 (95% CI = 0.635–3.964; *p* = 0.007), indicating that AgNO_3_ exhibited a higher tendency for toxicity than AgNPs in aquatic organisms. In terrestrial ecosystems, effect size = 6.283 (95% CI = 3.344–9.222; *p* = 0.000), indicating that AgNO_3_ had a significantly higher toxicity effect on terrestrial organisms. These findings suggest that the effects of silver are not uniform across different ecosystems, and that the toxicity of the ionic form is particularly pronounced in terrestrial environments. Figure 3 presents the results of the subgroup analysis by ecosystem type.

Another potential factor contributing to heterogeneity, exposure time, was also evaluated. For the toxicity tests, exposure time was divided into four range groups (1: 0–23 h, 2: 24–47 h, 3: 48–71 h, 4: 72+ h), and a subgroup analysis was conducted to assess its impact on heterogeneity.

Subgroup analysis across these groups yielded an overall effect size of 2.952 (95% CI = 0.113–5.790; *p* = 0.042), demonstrating a statistically significant effect of exposure time on the difference between AgNO_3_ TV and AgNP TV.

According to the exposure time, the effect size was found to be negative for 0–23 h (SMD = −0.531; 95% CI = −4.327–3.264; *p* = 0.784). The negative direction indicates that TVs of AgNO_3_ were higher than AgNPs in this time range, thus indicating that AgNPs exhibited a more toxic effect. The effect size was found to be positive and high for 24–47 h of exposure (SMD = 4.910; 95% CI = 2.194–7.627; *p* = 0.000), showing that AgNO_3_ was significantly more toxic compared to AgNPs. The effect size for 48–71 h of exposure was positive but not statistically significant (SMD = 1.795; 95% CI = −1.708–5.298; *p* = 0.315). At 72+ hours of exposure, the effect size was again found to be high and significant (SMD = 4.965; 95% CI = 1.756–8.173; *p* = 0.002).

**Figure 4:**
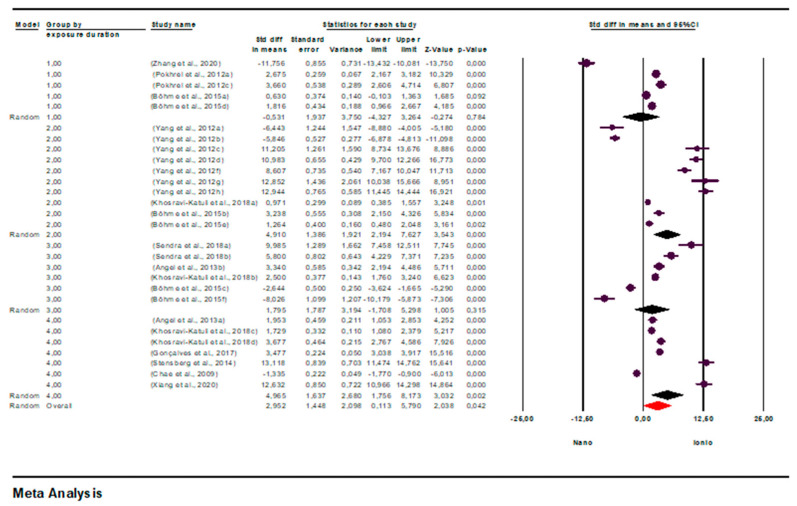
Subgroup analysis according to exposure time and forest plots (n = 28). CI = Confidence Interval; *p* = *p* < 0.05; Overall = Total mean size between studies; Nano = AgNPs TV; Ionic = AgNO_3_ TV.

These findings suggest that positive effect sizes indicate lower numerical TVs (and therefore higher toxicity) for AgNO_3_, while negative effect sizes indicate that AgNPs are more toxic. Overall, the results suggest that AgNO_3_ exhibits higher toxicity compared to AgNPs, particularly at 24–47 h and 72 h or longer of exposure (Figure 4).

Forest plot showing effect sizes (std diff in means; SMD) and 95% confidence intervals (CI) from studies comparing toxicity levels Nano = AgNP TV and Ionic = AgNO_3_ TV. Effect sizes were estimated with a random-effects model that considers between-study variation. Positive values indicate higher TV for nanoparticulate silver, thus indicating lower toxicity compared to ionic silver. Statistical significance was set at *p* < 0.05. CI = confidence interval; SMD = standardized mean difference; TV = toxicity value.

#### 3.3.3. Publication Bias Results

**Figure 5:**
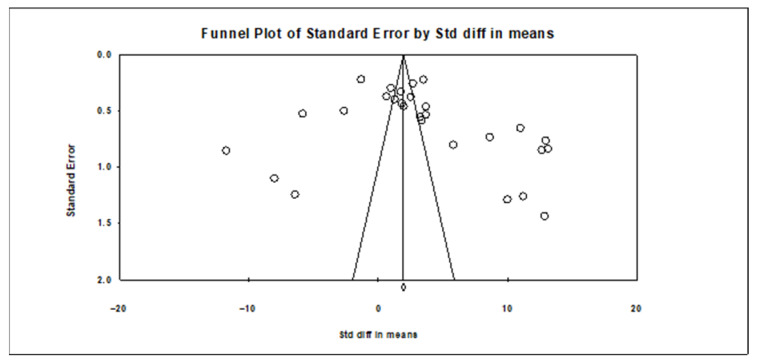
Funnel plot of studies.

The Kendall’s Tau coefficient, calculated according to the Begg and Mazumdar rank correlation statistics, was 0.25132, and the two-tailed *p*-value was 0.06054. This value, above the statistical significance level of 0.05, indicates that there was no significant finding indicating publication bias among the studies evaluated in the meta-analysis [50]. Thus, it is understood that there was no systematic bias in the data included in the study due to the reporting of the results, which supports the reliability of the findings. Furthermore, the assessment of publication bias in meta-analyses is not limited to statistical tests; the use of visual tools is also important. To this end, a funnel plot was prepared in the study and presented in Figure 5. The funnel plot contributes to a more transparent assessment of publication bias by showing the distribution of effect sizes according to sample sizes.

Additionally, a funnel plot was applied to visually assess publication bias. Also, the asymmetry of the funnel plot indicates possible publication bias in all included studies. This demonstrated the stability of the results of the meta-analysis. The funnel plot is shown in Figure 5. Slight asymmetry can occur due to the limited number of studies and the high heterogeneity of the dataset; the overall shape of the plot does not suggest strong publication bias. The statistical tests conducted also did not reveal any significant indication of bias, and the visual inspection of the funnel plot supports this finding.

Consequently, the statistical tests performed did not reveal any significant findings of publication bias, and the funnel plot was used to visually support these findings. Thus, the reliability of the meta-analysis results was demonstrated with both statistical and visual indicators.

## 4. Discussion

This meta-analysis compared the toxicity of the ionic and nano form of silver in microorganisms that live in various ecosystems by combining the results of independent studies. In this study, only ionic (AgNO_3_) and nanoparticulate (AgNPs) forms were compared; bulk silver was excluded from the analysis. The physicochemical properties reported in the included studies were examined, but since not all data contain sufficient detail in this respect, subgroup analyses were limited. Therefore, analysis of the underlying causes of toxicity has been limited to relying only on directly reported data, and generalizable conclusions about determining parameters such as ion release, surface properties, or particle size cannot be drawn. Although the number of studies included in the meta-analysis was limited, this was considered in the analysis design phase; random effects model and heterogeneity analyses (Q and I^2^ statistics) were used to account for differences between studies.

This meta-analysis compared the toxicity of the ionic and nano forms of silver in microorganisms that live in various ecosystems by combining the results of independent studies. The result of the meta-analysis showed that the overall mean effect size according to the standardized mean difference random-effects model was very strong, 3.268 (95% CI = 1.805–4.731; *p* = 0.000). The results suggest that the toxicity values (TV) of the nanoparticulate form of silver are statistically significantly higher than those of the ionic form. In this study, “TV” is used as an abbreviation for the general expression of dose–response curves derived from the included studies. The lower the values of EC50, LC50, and LD50 (collectively referred to as TV), the greater the toxicity of the substance [14,61]. This meta-analysis demonstrates that, for organisms across various ecosystems, ionic silver exhibits significantly higher toxicity compared to silver nanoparticles. Effective Concentration the EC50, LC50 and LD 50 values are a quantitative measure of the dose–response curve, which is widely used in toxicology. For example, the EC50 is the concentration of the test substance at which 50% of the population shows a response after a given exposure time. The lower the EC50 value, the lower the concentration of the test substance that elicited a toxic response [14,62]. This meta-analysis shows that ionic silver is more toxic than nanoparticulate silver, with a greater effect.

Comparing the toxicity of nano- and ionic forms of metals, meta-analysis study revealed that ionic metals are more toxic to terrestrial and aquatic ecosystems than nanometals [37]. On the other hand, there are studies showing that the ecotoxicity of nanomaterials is of the same order of magnitude as that of dissolved ions. Similarly to our study, in a meta-analysis study with a limited data set covering terrestrial and aquatic ecosystems, the toxicity rate of ionic silver is 0.00026 to 86.6 times greater than nanoparticulate silver [37]. Another study consistently compared the toxicity of nanoparticulate silver and ionic silver at the same concentrations and found that silver ions were more toxic than nanoparticulate silver and that the toxicity of nanoparticulate silver was mainly caused by the release of ionic silver [63]. In 2018, a comparative study of the toxicity of silver ions and silver nanoparticles in a microorganism species investigated the specific toxic effects of these two different forms of silver and found that they have a different toxicity relationship when present together [64]. Our findings are generally consistent with the existing literature: the terrestrial subgroup—while meeting all search and inclusion criteria—was largely informed by a single *C. elegans* study. For this reason, the evidence representing terrestrial ecosystems is inherently limited and should be interpreted with caution.

These studies have concluded that nanoparticulate silver toxicity on organisms should be re-evaluated with the ability to control ion release factors such as pH and ionic strength. Our study provided the bandwidth for toxicities in these reported studies, which allowed us to formulate meaningful conclusions as well as provide worst-case scenarios. This result, reinforced by meta-analysis, argues for a new horizon to produce nanoparticles with less toxicity and greater stability in the environment using different synthesis methods. The results indicate that ionic silver (AgNO_3_) generally exhibits greater toxicity than silver nanoparticles (AgNPs), as reflected in lower overall TV. Although the magnitude of this difference varies with exposure duration, AgNO_3_ maintains higher toxicity across most time points. This suggests that the observed difference in toxicity depends not only on the chemical form but also on the duration of exposure, and this variable must be taken into account in environmental risk assessments. This temporal pattern suggests that exposure duration plays a key role in the observed toxicological differences, likely due to the time-dependent dissolution and availability of silver ions.

In line with existing literature, this meta-analysis includes studies reporting both higher and lower toxicity of silver nanoparticles (AgNPs) relative to ionic silver (AgNO_3_). The dataset comprises studies that met strict inclusion criteria for quality and relevance, regardless of the direction of their findings. Notably, instances where AgNPs were found to be more toxic than AgNO_3_—contrary to the general trend—are reflected in the meta-analysis results as negative effect sizes, consistent with conventional reporting practices in ecotoxicological meta-analyses.

Since the biological properties of the organisms included in the study, such as incubation period and metabolic capacity, were not reported in a way suitable for statistical comparison at the species level, they could not be included in this analysis. To include such variables in the analysis, experimental studies need to adopt a more holistic data reporting standard. Systematic reporting of such biochemical parameters in future studies will enable more detailed meta-analyses on the mechanism of toxic effects.

Considering that exposure duration may be one of the underlying factors contributing to heterogeneity, a subgroup analysis was conducted based on different exposure times. This analysis revealed a temporal relationship in the comparative toxicity of AgNO_3_ and AgNPs based on toxicity values. At early exposure durations (≤24 h), silver nanoparticles (AgNPs) were found to be more toxic than ionic silver (AgNO_3_), as indicated by negative effect sizes. However, this trend reversed at longer exposure durations, where AgNO_3_ generally exhibited higher toxicity than AgNPs. Although the effect sizes did not follow a consistently increasing pattern over time, they remained predominantly positive, indicating a time-dependent shift in relative toxicity in favor of ionic silver. These qualitative classifications are only intended to interpret the observed trends. Analyses were conducted as subgroup comparisons based on five different exposure times (1: 0–23 h, 2: 24–47 h, 3: 48–71 h, 4: 72+ h). Although statistical power is limited in these analyses, they provide a meaningful basis for generating hypotheses about time-dependent trends. This finding should not be interpreted as the toxic effects of nanoparticles decreasing over time or that they have a healing effect. On the contrary, the early toxic effect observed in certain short-term experiments is related to the initial high ion release, reactive oxygen species production and rapid binding potential of AgNPs to the cell surface [65].

The higher toxicity of AgNPs at exposure times of ≤24 h in some studies may be attributed to the fact that these particles release more ions in a short time and penetrate the target cells quickly. This may be related to the decrease in dissolution kinetics and bioavailability of AgNPs at longer exposure times [66].

These findings agree with previous studies, which have also suggested that the toxicity of silver compounds can vary depending on exposure duration. Our results align with existing literature that highlights the importance of temporal factors in understanding the environmental risks of AgNO_3_ and AgNPs [67,68,69]. This consistency with earlier research further underscores the relevance of considering exposure time as a critical variable when assessing the toxicity of silver-based compounds.

The observed differences in toxicity can be explained by the faster dissolution of AgNO_3_ and its effective action in biological systems [6,70]. On the other hand, AgNPs tend to aggregate and dissolve over time, especially in aquatic environments [12,71]. Due to these properties, AgNPs initially have lower bioavailability, and their acute toxic effects decrease over time. These findings should be further supported by additional research. These dynamic differences help us understand how the toxicity of silver compounds in biological systems is influenced by their dissolution and bioavailability characteristics over time. The significance of this study lies in enabling a better understanding of the time-dependent effects of silver compounds’ toxicity. Additionally, such analyses emphasize the need to consider the time factor in environmental risk assessments and regulatory approaches. Exposure time is an important variable affecting the toxic effects of nanomaterials. However, analysis of such variables in meta-analyses is only possible if there is consistent and systematic data presentation in the literature. In this study, not only the effect of time on the direct toxic effect, but also how this relationship is shaped in terms of trends in the literature is evaluated. The differing effects of AgNO_3_ and AgNPs on ecotoxicology will play a critical role in shaping future environmental policies and management strategies.

This study, while providing a comparative assessment of the toxicity of ionic and nanoparticle silver forms, has some limitations stemming from the structure of the literature data used. Firstly, this meta-analysis includes multiple effect sizes derived from the same studies, reflecting different organisms, exposure durations or particle characteristics. Because these within-study effect sizes are not fully independent, the results should be interpreted with caution. All values were standardized to effect sizes to reduce structural imbalance: the non-independence of these observations is an inherent limitation of the dataset. The small number of studies did not allow for meaningful sensitivity analyses, and this constraint should be considered when interpreting the pooled effect sizes. Also, the inclusion of only eleven studies was a result of stringent inclusion criteria established for methodological consistency and data quality. Furthermore, the lack of consistent reporting across studies of important physicochemical properties that may influence toxicity, such as particle size, surface coverage, solubility ratio, and ion release, prevented subgroup analyses based on these variables. Because most existing studies lacked a standardized statistical reporting structure, some parameters could not be evaluated with advanced analysis methods such as meta-regression. However, these limitations do not eliminate the validity of the study findings; on the contrary, they point to the need for improved reporting standards for future research in environmental toxicology.

## 5. Conclusions

This research provides a systematic quantitative assessment of the available literature through meta-analysis, quantitatively comparing the toxicity, sensitivity and statistical power of ionic and nanoparticulate forms of silver for various organisms. Also, this meta-analysis study provides a holistic database that can be used in environmental risk assessment studies by comparatively evaluating the toxicity levels of silver forms. The findings obtained can provide preliminary information to policy makers, especially in terms of determining sensitive species, effects of exposure duration, and comparative interpretation of toxic effects.

This approach provides a detailed overview of the expected toxicity of nanoparticles and dissolved forms of silver metal to ecosystem components under specific conditions. Only these forms were considered within the scope of the analysis; physicochemical mechanisms such as solubility behavior, ion release, and reactive oxygen species production could not be directly evaluated due to data limitations. On the other hand, it does not provide any new insights into the chemical and physical properties of silver in nano or dissolved form, the mechanism of toxicity, the degree of dissolution of a metal particle, or the sensitivity of species in the ecosystem.

This study, focusing on numerical data, highlights that this biodiversity may partially explain the variability in toxic responses. Since such parameters should be included in all studies at a certain data quality to be evaluated in meta-analysis, we believe that if such data are reported more regularly in the future, it will be possible to directly include these variables in the analyses. Additionally, although toxicity databases provide a rich source of environmental data, the lack of standardized statistical reporting currently limits their use in meta-analytic studies. Properties such as particle size, surface area, and zeta potential could not be analyzed directly as independent variables in the meta-analysis. Future improvements in database reporting may increase the scope of such analyses. Although particle size was not directly included as a variable in our analysis, the effect of these physicochemical differences was indirectly reflected. However, due to limitations in the data structure, no advanced statistical modeling (e.g., meta-regression) was performed for variables such as particle size. As a result of this study, a minimum reporting requirement is recommended to increase the methodological transparency and comparability of future comparative toxicity studies. In this context, studies are recommended to present basic nanoparticle characterization information (size distribution, surface coverage, zeta potential, and dissolution/ion release data), clearly specify experimental conditions (pH, temperature, exposure time, and medium composition), and statistical reporting elements (sample size, variance measures, and dose–response curve parameters) in a standardized format. Such a reporting standard will strengthen the methodological harmonization of future studies and contribute to more reliable comparative toxicity assessments of ionic silver and nanosilver.

The increasing presence of nanomaterials in many areas of life necessitates an overall assessment of their environmental and toxicological impacts. This study systematically brings together existing research to provide more consistent and comparable results regarding the effects of nanomaterials and ionic forms. The findings provide an important basis for strengthening environmental risk assessments and enabling decision-makers to develop more reliable measures. Given the future trends in the use of nanomaterials, continuing such meta-analytical approaches will contribute to reducing scientific uncertainties and supporting sustainable management policies.

## Figures and Tables

**Table 1:** Overview of experimental parameters included in meta-analysis.

Study Name	Sample Size	Organism	Exposure Time	Ecosystem	Test Conditions
Zhang et al., 2020 [52]	1	*Escherichia coli*	3 h	aquatic	T = 25 °C pH = 6.8
Sendra et al., 2018 [53]	2	*Chlorella autotrophica*, *Dunaliella salina*	48 h	aquatic	T = 20 °C pH = 7
Angel et al., 2013 [54]	2	*Pseudokirchneriella subcapitata*, *Ceriodaphnia dubia*	72 h	aquatic	T = 24 °C
Pokhrel et al., 2012 [55]	2	*Escherichia coli*	5 h	aquatic	T = 35 °C pH = 7.2
Yang et al., 2012 [2]	7	*Caenorhabditis elegans*	24 h	terrestrial	T = 22 °C pH = 8.3
Khosravi-Katuli et al., 2018 [56]	4	*Cyprinus carpio*	24 h, 48 h, 72 h, 96 h	aquatic	T = 21.1 °C pH = 7.3
Gonçalves et al., 2017 [57]	1	*Physa acuta*	96 h	aquatic	T = 22 °C pH = 7.9
Steffi et al., 2015 [58].	6	*Danio rerio*	2 h, 26 h, 71 h	aquatic	T = 26 °C pH = 7.6
Stensberg et al., 2014 [59]	1	*Daphnia magna*	72 h	aquatic	T = 22 °C pH = 8.2
Chae et al., 2009 [6]	1	*Oryzias latipes*	96 h	aquatic	T = 25 °C pH = 7.5
Xiang et al., 2020 [60]	1	*Cyprinus carpio*	96 h	aquatic	T = 18 °C pH = 7.1

## Data Availability

The original contributions presented in this study are included in the article. Further inquiries can be directed to the corresponding author.

## Abbreviations

The following abbreviations are used in this manuscript:

AgNPs	Silver nanoparticles
AgNO_3_	Silver nitrate
Ag^+^	Ionic silver
CI	Confidence Interval
CMA	Comprehensive Meta-Analysis
EC50	Half maximal effective concentration
h	Hour
LC50	Lethal Concentration 50
LD50	Lethal Dose 50
n	Sample size
*p*	Probability value
pH	Potential of Hydrogen
SMD	Standardized mean difference
t	temperature
TV	Toxicity values
